# Supplementary material for: Cation-controlled wetting properties of vermiculite membranes and its promise for fouling resistant oil–water separation
Source: Nat Commun. 2020 Feb 27;11:1097. doi: 10.1038/s41467-020-14854-4 (PMC7046718; doi:10.1038/s41467-020-14854-4)
Supplement: Supplementary file 1 — Supplementary Information [file 41467_2020_14854_MOESM1_ESM.pdf]

Supplementary Information

Cation controlled wetting properties of vermiculite membranes and its promise for fouling resistant oil-water separation

Huang *et al*.

Supplementary Figures

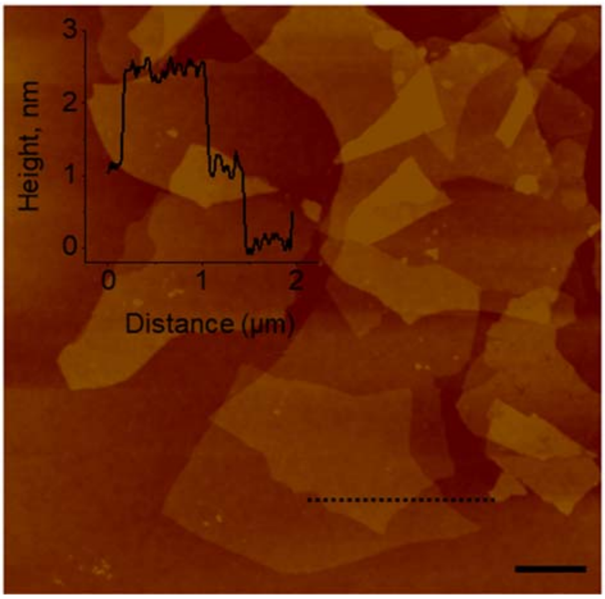

Supplementary Figure 1| AFM characterisation. AFM image of vermiculite flakes drop-casted on a silicon wafer. Inset: Height profile along the dotted line showing vermiculite flakes has an average thickness of ≈1.5 nm. Scale bar, 750 nm.

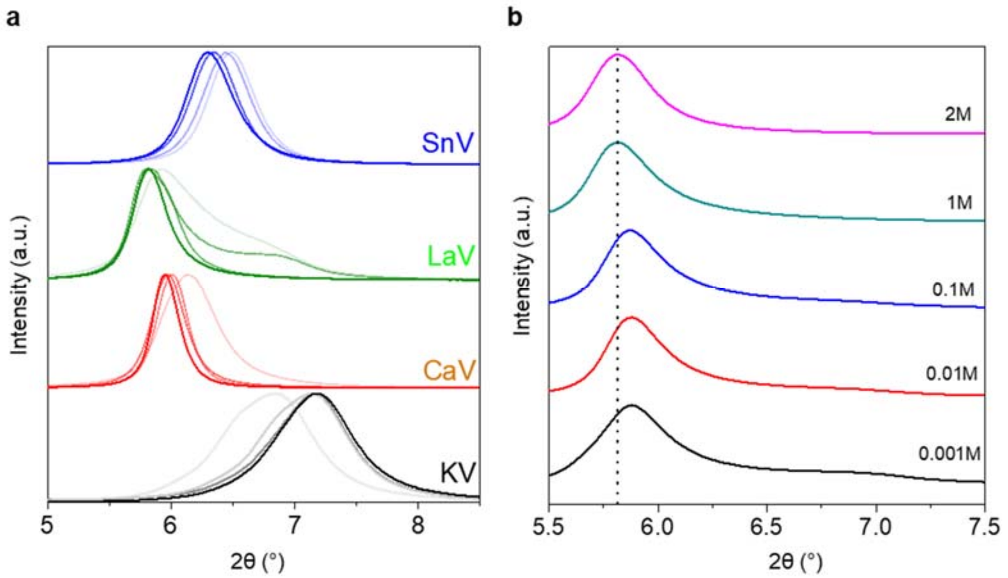

Supplementary Figure 2| XRD characterisation of ion exchange in V-laminates. a, Evolution of the XRD of V-laminates as a function of ion-exchange time. XRD after exposing the laminates to the corresponding 1M salt solution for 20, 40, 60, and 120 minutes are shown. Light to dark colour of the spectra indicates the exposure time from 20 to 120 minutes. Source data are provided as a Source Data file. **b**, XRD taken after exposing the LiV laminate to $LaCl_3$ solution with different concentrations for 1 hour. The dotted line indicates the peak position after the complete exchange of Li with La. From both the figures it can be concluded that immersing the V-laminates in 1M salt solution for 1 hour is sufficient for the full ion exchange process. Source data are provided as a Source Data file.

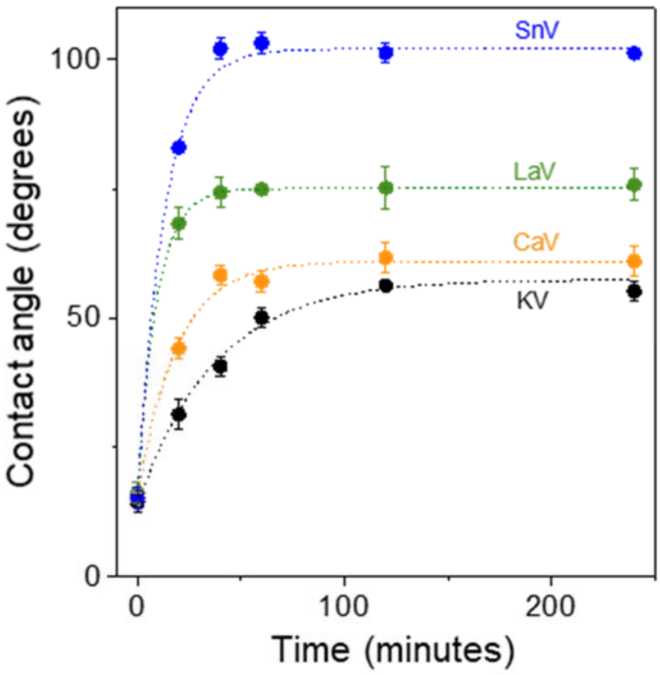

Supplementary Figure 3| Change in contact angle during the ion exchange process. Contact angle as a function of the corresponding 1M salt solution exposure time (methods) during the ion exchange process. Source data are provided as a Source Data file.

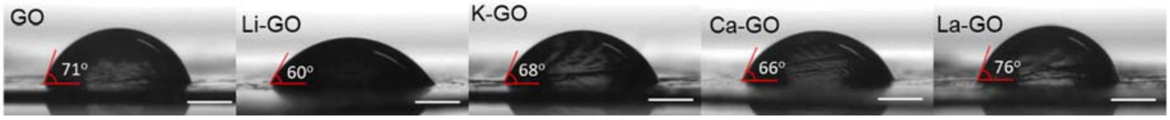

Supplementary Figure 4| Contact angle of cation modified graphene oxide (GO) membranes. Water contact angle of pristine GO and Li, K, Ca, and La modified GO membranes. Scale bar, 750 µm.

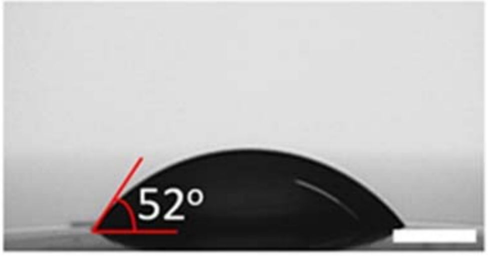

Supplementary Figure 5| Wetting properties of NaV laminate. Water contact angle of NaV laminate in ambient condition. Scale bar, 750 µm.

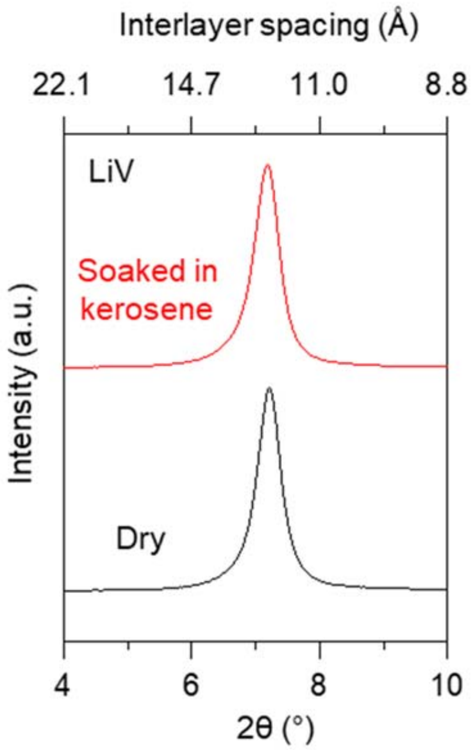

Supplementary Figure 6| Non-swelling Vermiculite laminate in oil. X-ray diffraction (XRD) from a vacuum dried free-standing LiV-laminate and the same membrane soaked in kerosene for 48 h (colour coded labels). Source data are provided as a Source Data file.

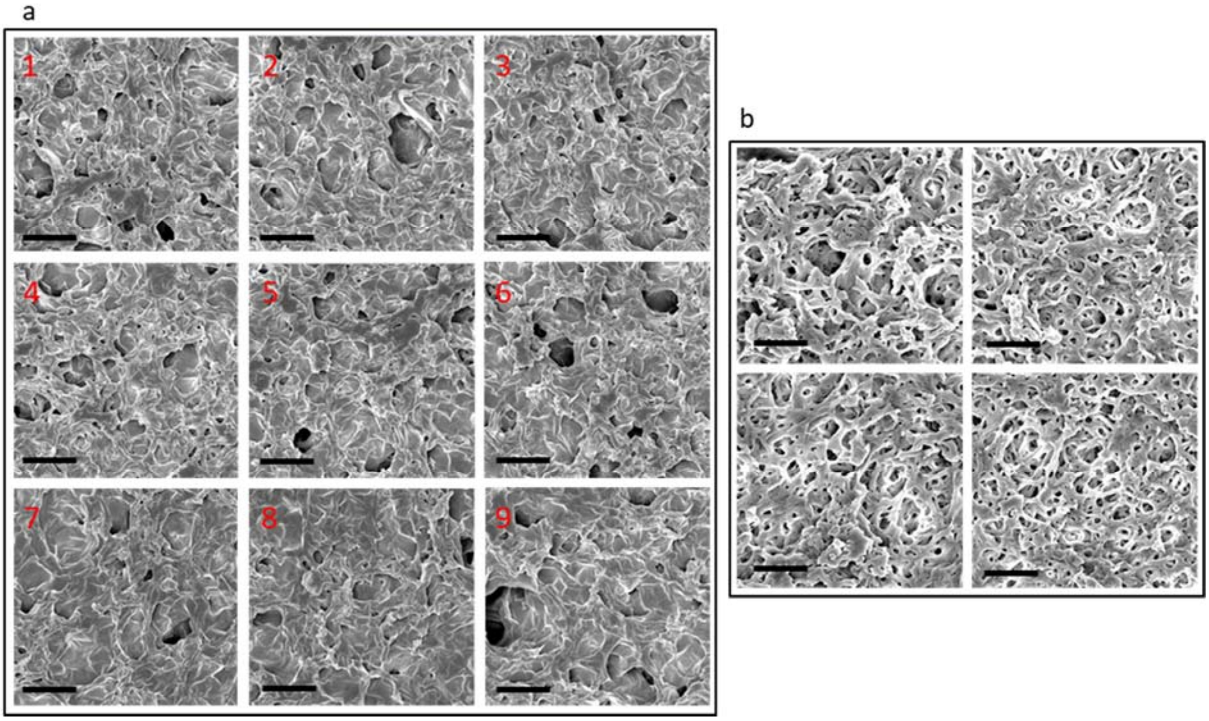

Supplementary Figure 7| SEM characterisation of LiV coated PA membrane. a, Random SEM images of the 30 nm LiV coated PA membrane from 9 different positions. **b**, Random SEM images of a bare PA membrane. Scale bars, 1 µm.

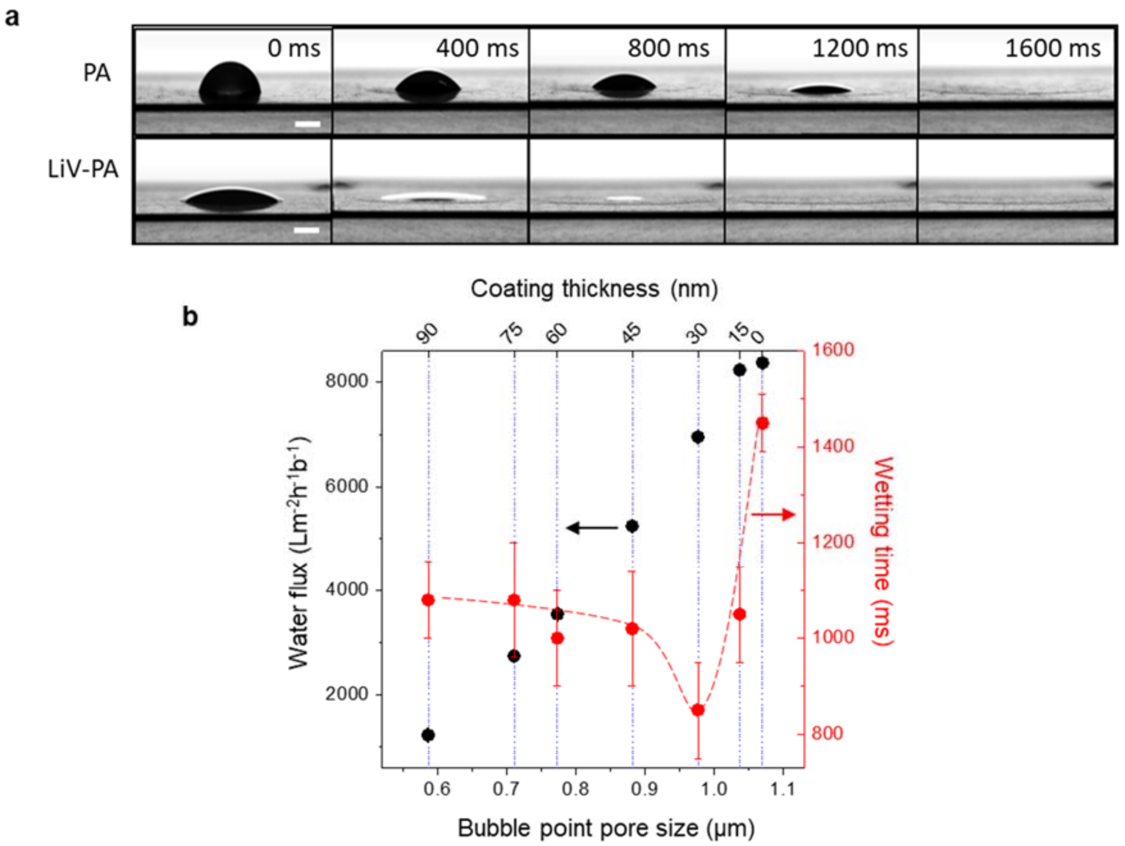

Supplementary Figure 8| Water wetting and water flux through LiV coated polyamide (PA) membrane. a, Water wetting behaviour of the PA and LiV coated (30 nm) PA membranes evaluated by contact angle measurements in dynamic mode. Scale bars; 1000 µm **b**, Water flux and water wetting time for LiV coated PA membranes with various coating thickness or pore size (colour coded axis). The dashed line is a guide to the eye. Error bars denote standard deviations using five different measurements. Water flux was measured by filtering 200 ml of water (after reaching into a steady state flux by filtering water for more than one hour) using a dead-end pressure filtration system at a pressure of 1 bar. The high-water flux and low wetting time of 30 nm LiV coated PA membranes makes them as a choice for antifouling studies. Source data are provided as a Source Data file.

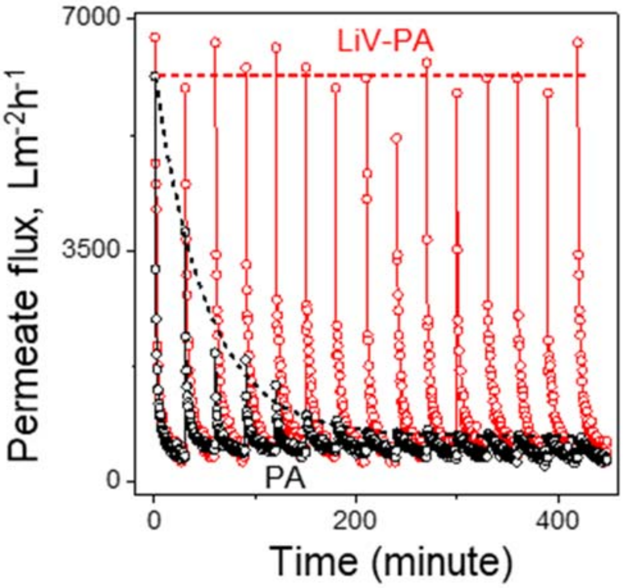

Supplementary Figure 9| Cyclic emulsion separation. Permeate flux as a function of time during the multiple cycle emulsion separation by dead-end filtration at a pressure of 1 bar. The dotted lines are guide to eye for the initial permeate flux at each filtration cycle. The decrease in the permeate flux with time in each cycle is due to the oil droplet deposition on the surface of the membrane. These droplets were easily removed by water rinsing after each cycle in the case of LiV coated membrane whereas it fouls the bare PA membrane permanently. Source data are provided as a Source Data file.

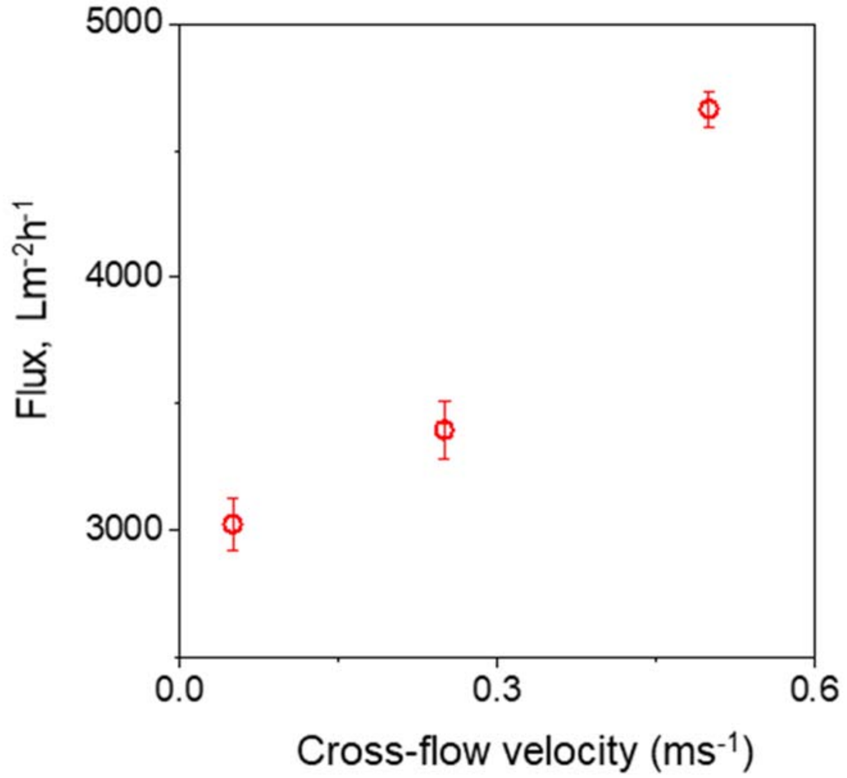

Supplementary Figure 10| Cross-flow emulsion separation. Steady-state permeate flux of LiV coated PA membrane as a function of cross-flow velocity during the emulsion separation at 1 bar pressure. Source data are provided as a Source Data file.

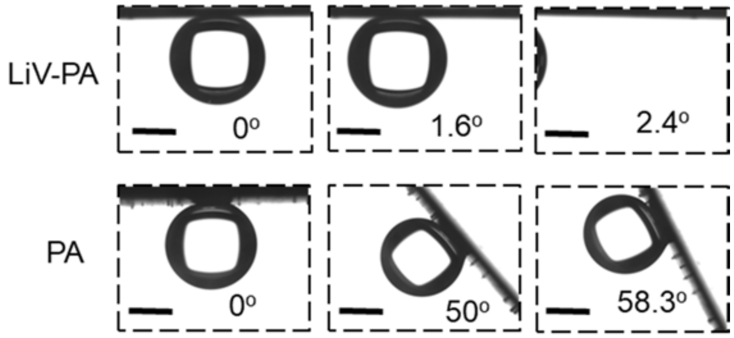

Supplementary Figure 11| Oil droplet roll-off angle. Photographs of an oil droplet (kerosene, 10 µL) on the surface of LiV coated polyamide (LiV-PA) and bare polyamide (PA) membrane at different tilt angles. Scale bars, 1 mm. For the LiV-PA membrane, at 1.6^{o} the droplet starts to slide and completely rolls off at 2.4^{o} whereas, for the bare PA, the droplet starts to slide at 50° and completely rolls at 58.3^{o}. The negligibly small roll-off angle for LiV-PA surface implies a remarkably weaker adhesion force between the oil droplet and the membrane surface.

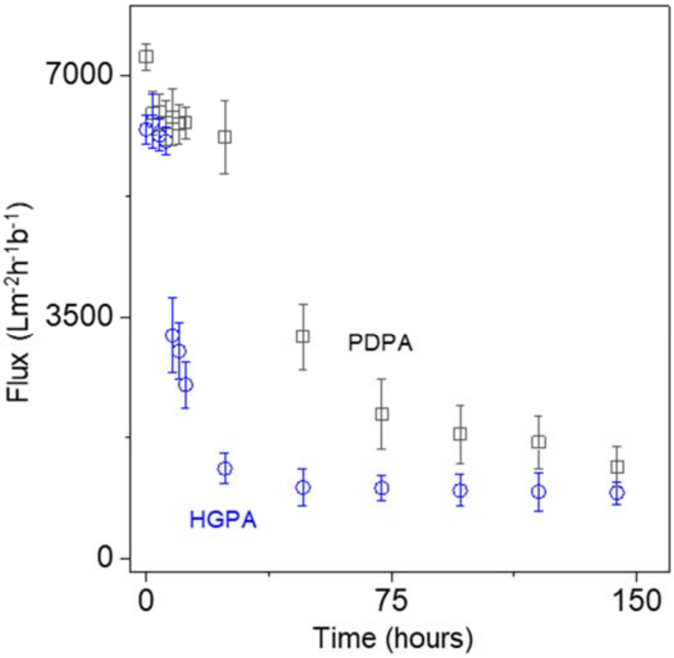

Supplementary Figure 12| Fouling in superhydrophilic polymer-coated PA. Water flux through polydopamine coated PA (PDPA) and polyacrylamide hydrogel coated PA (HGPA) as a function of time during which the membrane was in contact with the oil. The measurement of the flux is performed at 1bar differential pressure. Error bars denote standard deviation using five different measurements. Source data are provided as a Source Data file.

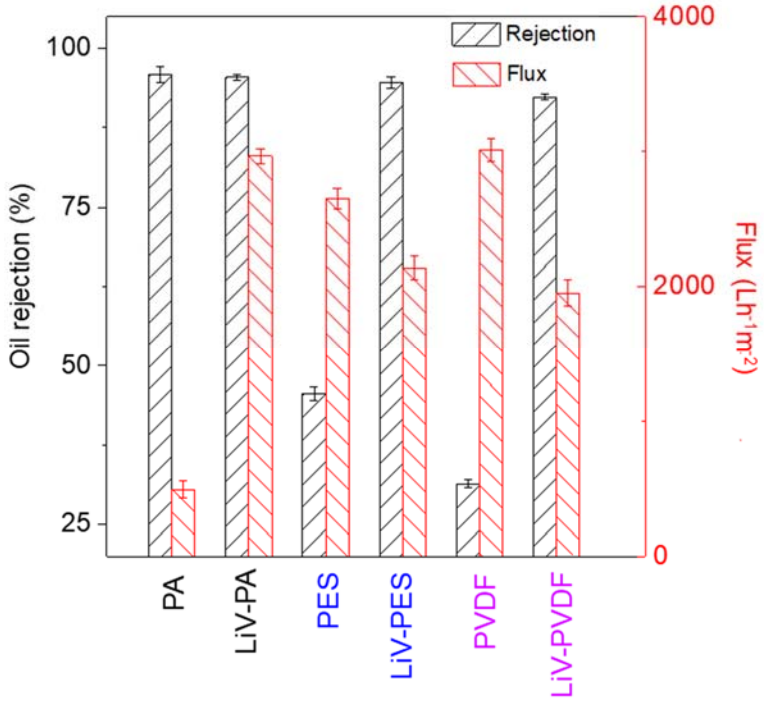

Supplementary Figure 13| Emulsion separation performance of LiV coated microfiltration membranes. Oil rejection and steady state permeate flux for polyamide (PA), polyethersulfone (PES), and polyvinylidene difluoride (PVDF) before and after LiV coating. The enhanced oil rejection for LiV-PES and LiV-PVDF compared to bare PES and PVDF is due to the increase in underwater oleophobicity after LiV coating (Supplementary Figure 12). The test was carried out with 0.05 ms^{-1} cross-flow speed at 1 bar pressure. For PA and PVDF, 30 nm LiV coating was used whereas for PES due to its smooth surface topography, 5 nm coating was sufficient. Increasing the coating thickness leads to a significant reduction in flux. PES and PVDF (PVDFV0.2) membranes with a pore size of 0.22 µm were purchased from Merck Millipore and Sterlitech, respectively. Source data are provided as a Source Data file.

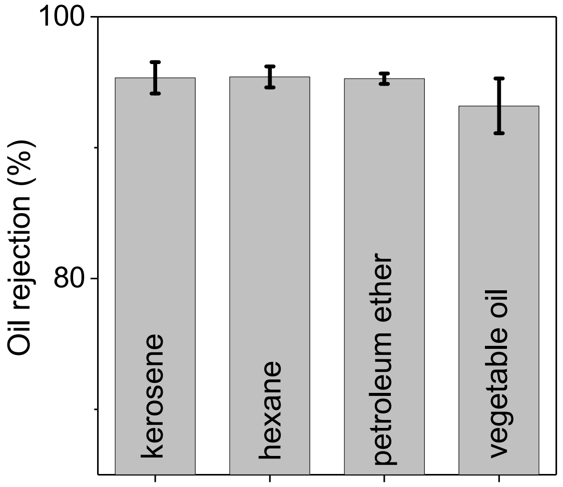

Supplementary Figure 14| Emulsion separation. Oil rejection of the LiV coated PA membrane for different types of emulsion prepared from different oils. Error bars denote standard deviations using three different samples. Source data are provided as a Source Data file.

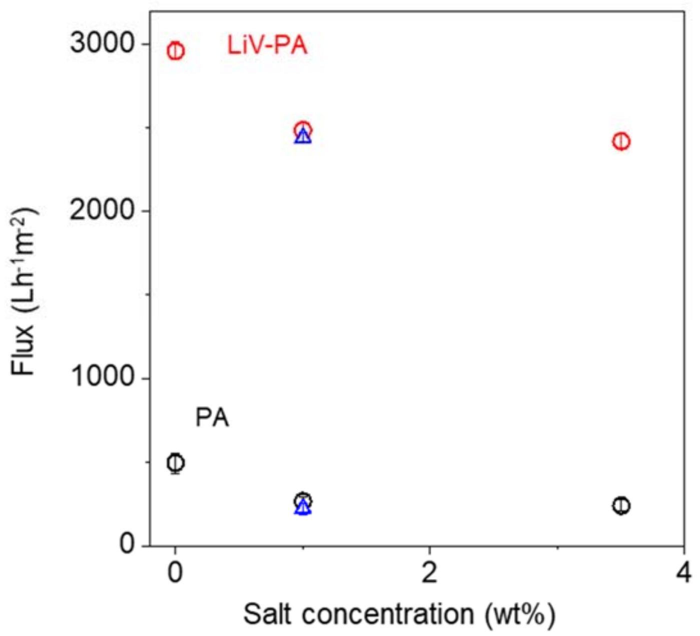

Supplementary Figure 15| Influence of salt on the emulsion separation. Steady-state permeate flux for LiV coated polyamide (LiV-PA) and bare polyamide (PA) as a function of NaCl salt concentration in the feed emulsion (colour coded labels). Blue coloured data points show the permeate flux for 1wt% KCl salt in the feed emulsion. A small decrease in the flux was observed after adding the salt for both LiV-PA and PA. The test was carried out with 0.05 ms^{-1} cross-flow speed at 1 bar pressure. Source data are provided as a Source Data file.

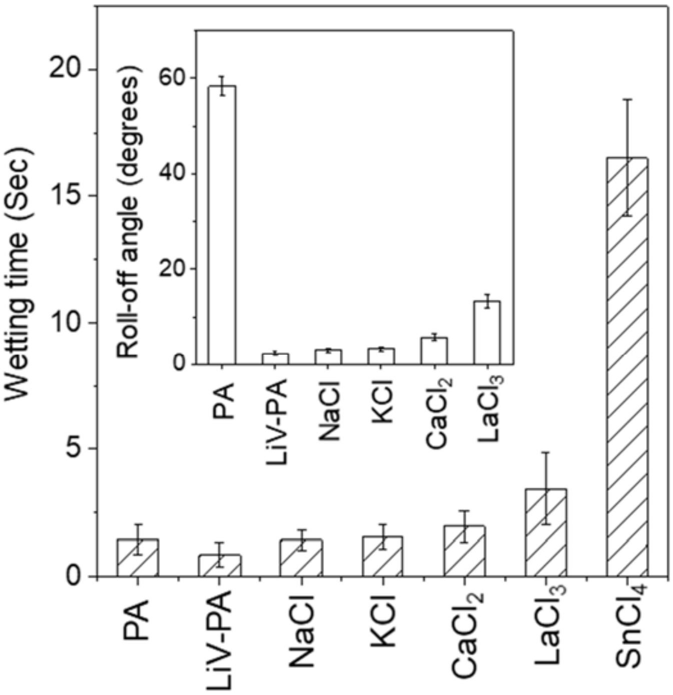

Supplementary Figure 16| Influence of exposure to salty emulsion on the wetting time of LiV coated PA. Water wetting time for LiV coated polyamide (LiV-PA) membranes exposed to different salty emulsions solutions of concentration one molar for 12 hours. Wetting time for reference polyamide and LiV-PA are also plotted. Inset: Oil droplet roll-off angle for LiV-PA membranes exposed to different salty emulsions solutions of concentration one molar for 12 hours. Source data are provided as a Source Data file.

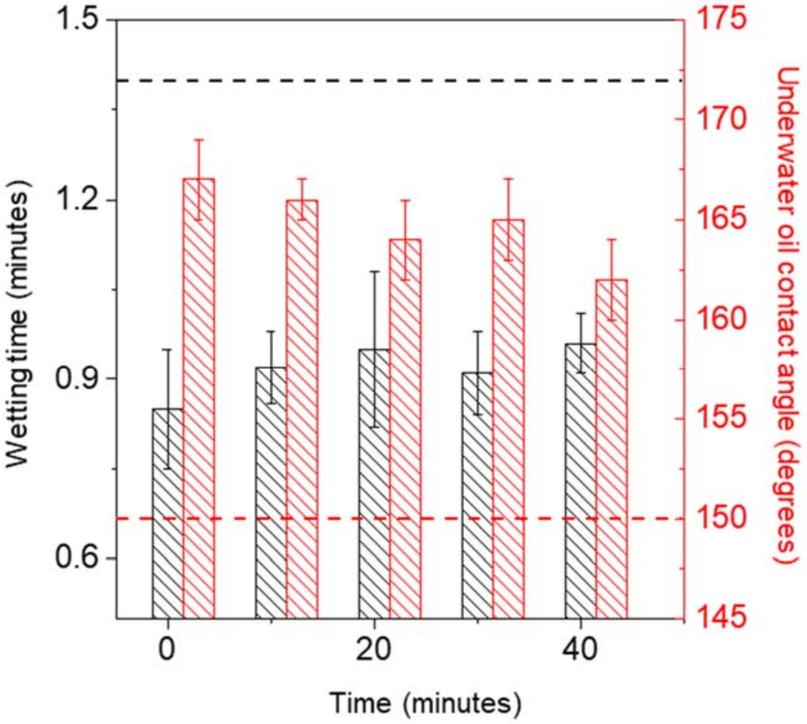

Supplementary Figure 17| Stability of LiV coating on PA. Water wetting time and underwater oil contact angle of LiV coated PA membrane measured after the direct water flushing with a speed of 5 $Lmin^{-1}$ for different intervals of time (colour coded axis). Black and red dashed line indicates the water wetting time and underwater oil contact angle of bare PA membrane. Error bars denote standard deviations using five different measurements. Source data are provided as a Source Data file.

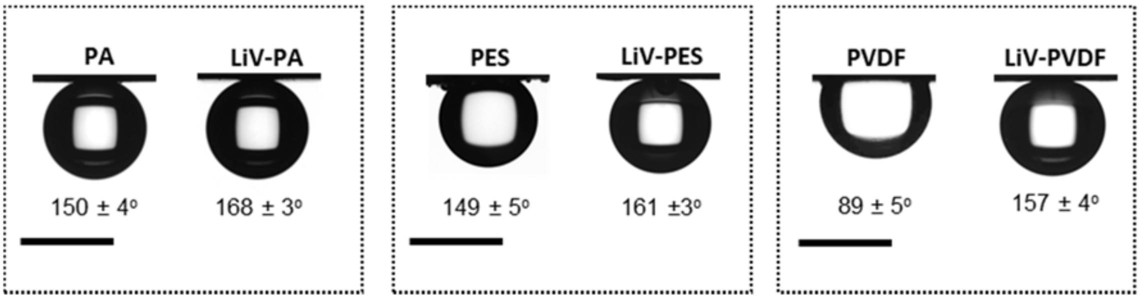

Supplementary Figure 18| Underwater oleophobicity. Underwater oil contact angle measured on bare and LiV coated polyamide (PA), polyethersulfone (PES), and polyvinylidene difluoride (PVDF). Scale bar; 1.5 mm. The coating thickness was 30 nm for PA and PVDF whereas 5 nm for PES.

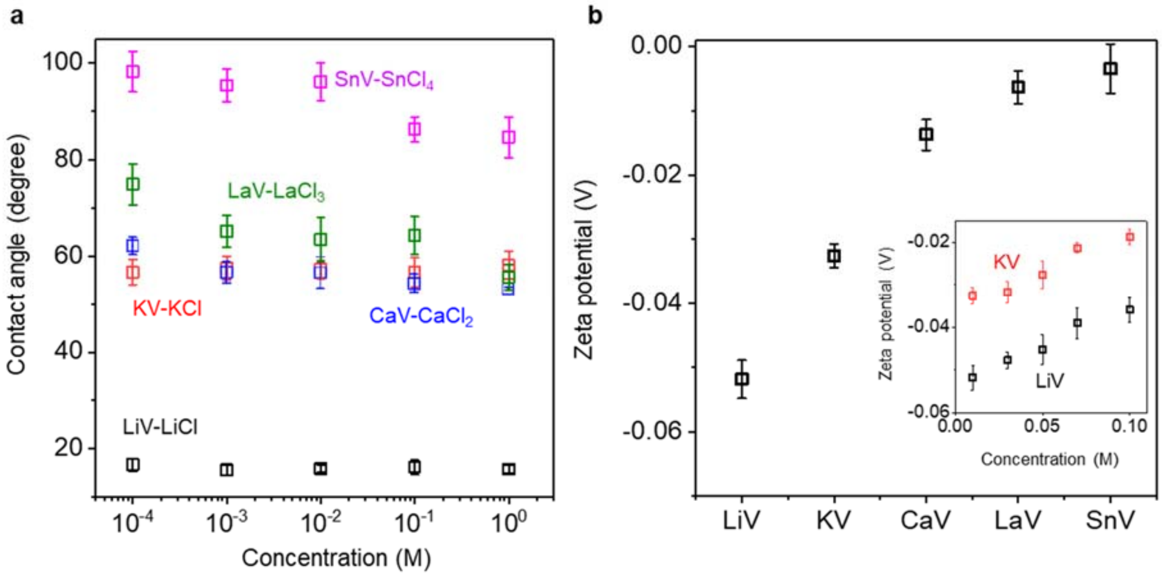

Supplementary Figure 19| Effect of ionic strength on contact angle and zeta potential. a, The contact angle as a function of ionic strength of the solution for various V-laminates. Source data are provided as a Source Data file. **b**, Zeta potential obtained for various V-laminates. Inset: variation of zeta potential as a function of ionic strength of the solution for KV- and LiV-laminates. Source data are provided as a Source Data file.

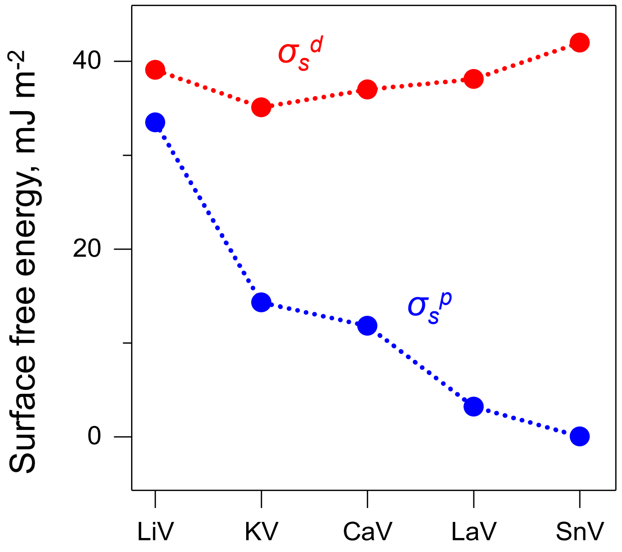

Supplementary Figure 20| Surface free energy for different V-laminates. Dispersion ($\sigma_S{}^d$) and polar ($\sigma_S{}^p$) components of the surface free energy for various vermiculite laminates estimated from the contact angle data. Source data are provided as a Source Data file.

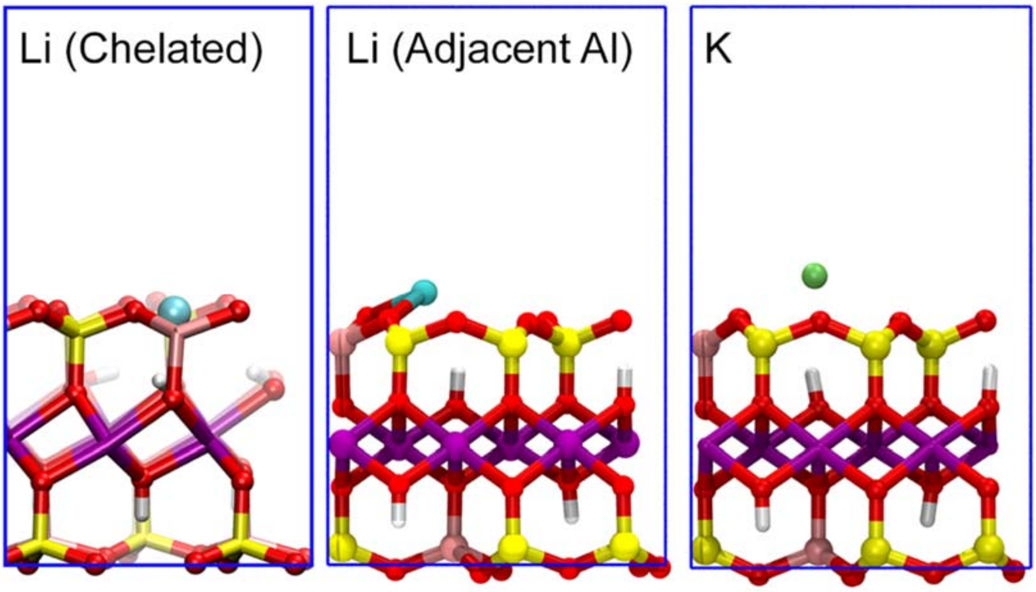

Supplementary Figure 21| Ion binding configurations. Showing two binding configurations for lithium and potassium ions. The chelated configuration for potassium is not found to be stable, this is likely due to the significantly larger ionic radius of the potassium cation. Oxygen atoms are shown in red, hydrogen in white, silicon in yellow, aluminium in pink, magnesium in purple, and lithium and potassium in cyan and green, respectively.

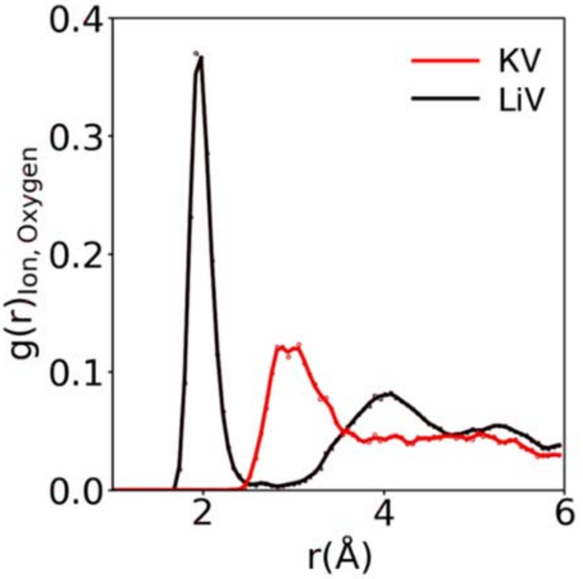

Supplementary Figure 22| Ion-Oxygen Radial Distribution Functions. Radial distribution functions (RDF) averaged over independent trajectories. The first peak in the lithium RDF corresponds to the hydration of the lithium ions bound to oxygen atoms adjacent aluminium dopants. The second, broader peak corresponds to water molecules hydrating the chelated lithium ions. Source data are provided as a Source Data file.

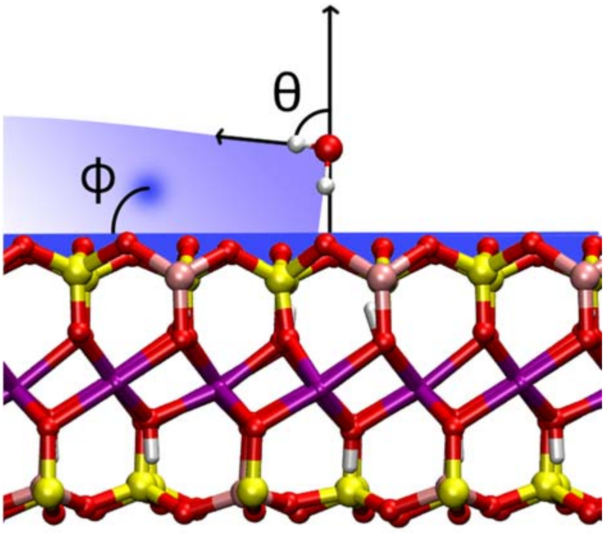

Supplementary Figure 23| Angular Definitions. Illustration of angular definitions for water molecules above the vermiculite surface. θ is defined as the angle between the vermiculite surface normal and water O-H bonds. ϕ is defined as the angle between the plane through the water molecule and the basal plane of the vermiculite surface.

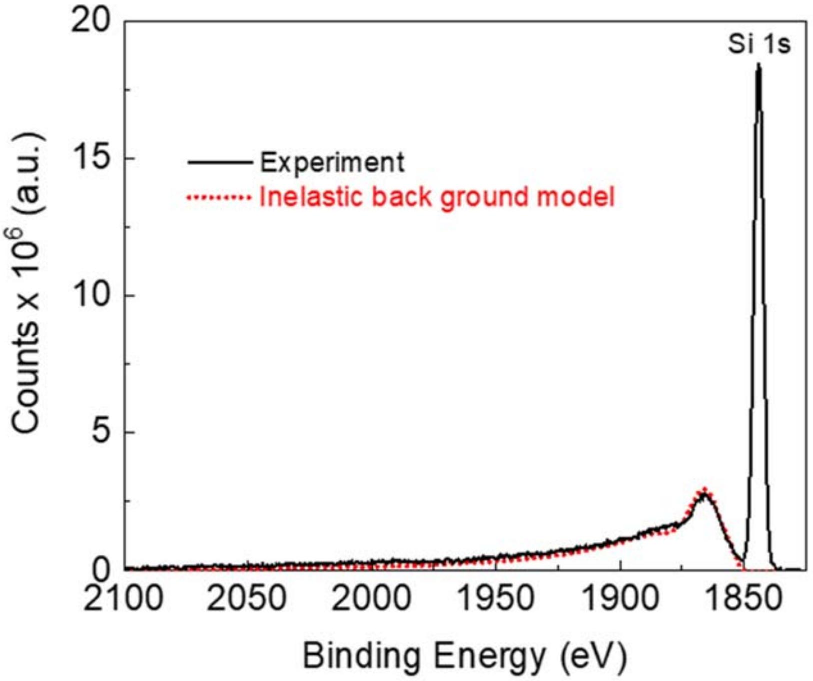

Supplementary Figure 24| HAXPES on 40 nm LiV coated PA . XPS of Si 1s core level (black line) and inelastic background model using the QUASES-Tougaard software, where an inelastic mean path of 14.6 nm is obtained using the TPP-2M formula (Ref. 41 in the main text). Source data are provided as a Source Data file.

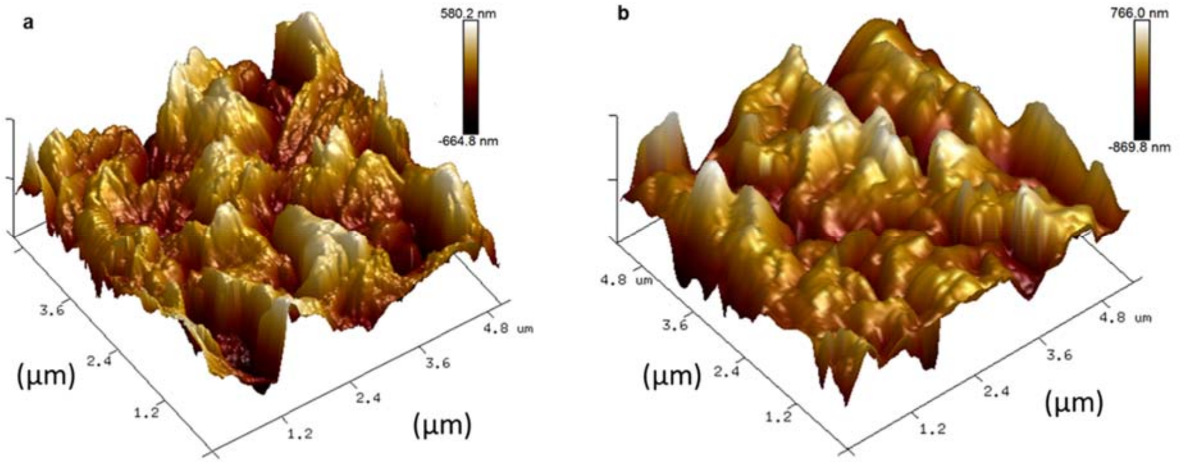

Supplementary Figure 25| Root mean square (RMS) roughness of the membranes. Atomic force microscopy (AFM) image of bare PA (a) and 30 nm LiV-PA (b). AFM Images were captured using a Bruker Dimension FastScan AFM operating in peak force tapping mode. The average RMS roughness obtained from three different samples of bare PA membrane and LiV coated polyamide membrane is 324 ± 112, and 376 ± 58, respectively.

Supplementary Table 1| ICP Analysis. Concentration of cations in various vermiculite laminates. BDL denotes 'below detection limit'. Within a factor of ~ 2-3, we did not notice any significant change in the cation concentration with different laminate. Hence, we believe the quantity of cations in different vermiculite laminate is similar and does not influence the observed wetting properties.

Moles mg^{-1}	LaV	KV	LiV	CaV	SnV	Bulk Vermiculite
K	1.3×10^{-7}	8.6×10^{-7}	2.7×10^{-7}	7.6×10^{-8}	1.7×10^{-7}	9.6×10^{-7}
Li	BDL	BDL	1.4×10^{-6}	BDL	BDL	BDL
Ca	BDL	BDL	BDL	6.8×10^{-7}	BDL	3.5×10^{-8}
La	4.5×10^{-7}	BDL	BDL	BDL	BDL	BDL
Sn	BDL	BDL	BDL	BDL	6.7×10^{-7}	BDL

Supplementary Table 2 | Contact angles. Water and diiodomethane Contact angle of different V-laminates. Error denote standard deviations using three different measurements.

Laminates	θ_{water}(°)	$\theta_{diiodomethane}$(°)
LiV	15±1	41±2
KV	56±2	38±3
CaV	63±3	45±2
LaV	75±2	43±2
SnV	101±2	35±3
